# Gastric IgG4-Related Autoimmune Fibrosclerosing Pseudotumour: A Novel Location

**DOI:** 10.5402/2011/873087

**Published:** 2010-11-07

**Authors:** Katie E. Rollins, Samir P. Mehta, Maria O'Donovan, Peter M. Safranek

**Affiliations:** ^1^Department of Upper Gastrointestinal Surgery, Addenbrooke's Hospital, Hills Road, Cambridge, CB2 0QQ, UK; ^2^Department of Histopathology and Cytology, Addenbrooke's Hospital, Cambridge, CB2 0QQ, UK

## Abstract

We describe the first reported case of an IgG4-related autoimmune fibrosclerosing pseudotumour located in the stomach of a 75-year old woman presenting with weight loss and vomiting. A lesion was detected in the gastric body at endoscopy. Subsequent characterisation by CT was suggestive of a gastrointestinal stromal tumour. Following laparoscopic resection, the patient recovered uneventfully. Histological examination of the resected specimen revealed an IgG4-related fibrosclerosing pseudotumour, a novel location for this histopathological entity.

## 1. Introduction

IgG4-related autoimmune fibrosclerosing pseudotumour was first reported to be found in various organs of patients with autoimmune pancreatitis (AIP), a form of chronic sclerosing pancreatitis associated with an infiltration of T cells and IgG4-expressing plasma cells. There are multiple recognised clinical manifestations of IgG4 sclerosing disease including sclerosing cholangitis, cholecystitis, retroperitoneal fibrosis, interstitial pneumonia, tubulointerstitial nephritis-and inflammatory pseudotumour [1]. We present the case of a patient with a gastric midbody mass which was characterised histologically as an IgG4-related autoimmune fibrosclerosing pseudotumour.

## 2. Case Report

**Figure 1:**
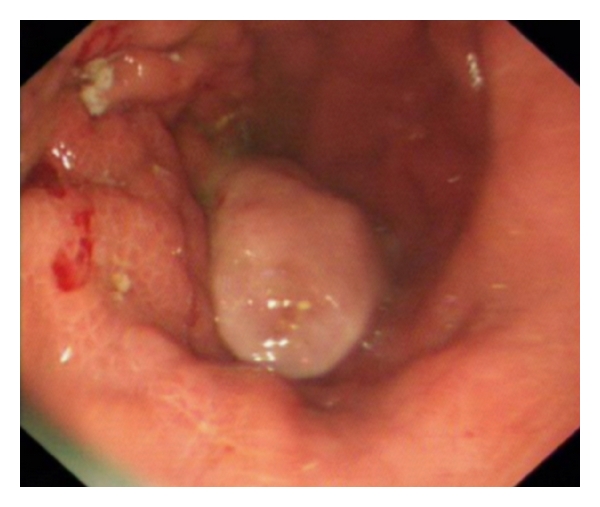
Oesophagogastroduodenoscopy (OGD). The large polypoid mass seen in gastric midbody.

**Figure 2:**
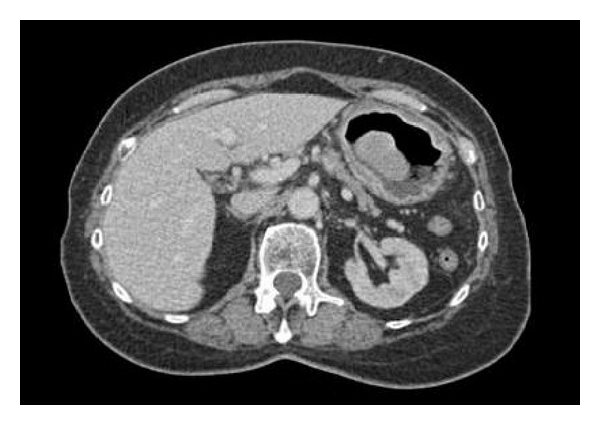
Computed tomography scan. Gastric mass arising from medial wall of stomach.

A 75-year-old woman presented to the fast track endoscopy service with a two-month history of vomiting, weight loss, and anaemia. She had no previous medical history of note and was independent. Initial gastroscopy revealed a large polypoid lesion in the gastric body (Figure 1) with abnormal appearing mucosa in the fundus, antrum, and duodenum. Multiple gastric biopsies were taken, which under microscopy showed markedly hyperplastic mucosa with evidence of chronic ulceration; there was no evidence of malignancy. Computerised tomography (CT) revealed a well-defined homogenous mass measuring 5 × 5.6 cm projecting within the lumen, arising from the medial wall of the body of the stomach (Figure 2). Two mesenteric lymph nodes measuring 9 mm and 6 mm and one enlarged left gastric lymph node were also identified. An endoscopic ultrasound was attempted but failed because the patient was unable to be intubated. 

The patient subsequently went on to have a laparoscopic resection of the lesion from the posterior aspect of the lesser curvature. This was performed by making an anterior gastrotomy and resecting the lesion using a laparoscopic linear stapler. Following an intraluminal bleed which was managed conservatively, she made an uneventful recovery and was discharged home on day 10 postoperatively.

**Figure 3:**
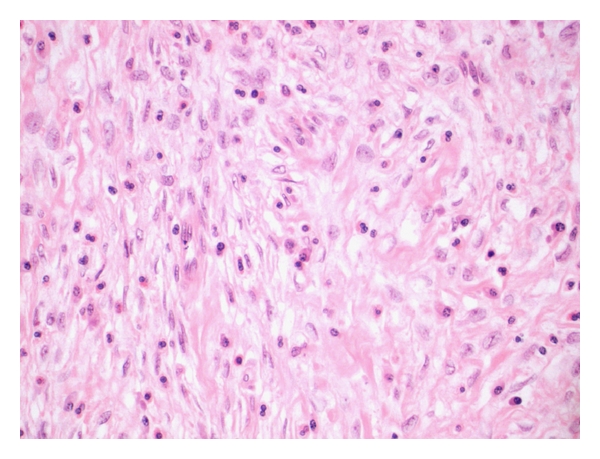
Histopathological analysis at 40x. Haematoxylin and eosin stain showing plump spindle cells set in a fibrous stroma with eosinophilic material and admixed inflammatory cells.

Histological examination of the specimen (Figure 3) showed interweaving fascicles of spindle cells, set in a fibrous stroma of abundant dense eosinophilic material and a prominent inflammatory cell infiltrate including lymphocytes, scattered plasma cells, and many eosinophils. There was one mitosis per fifty high-power fields. Immunohistochemical stains were negative for DOG 1, CD117, S100, desmin, ALK, and cytokeratin. Actin was equivocal and CD34 was positive in the spindle cell population. EMA and IgG4 stained the plasma cells, suggesting an IgG4-related autoimmune fibrosclerosing pseudotumour. There was an average of 39 IgG4-positive lymphoplasmacytic cells per high-power field. This lesion extended to the resection margin. The lymph nodes detected on CT which were removed with the specimen were reactive in nature.

The patient was seen in clinic two weeks later, by which time she had made a full recovery after surgery. Subsequent blood tests revealed that her serum IgG4 was within normal range (1.09); however, levels had not been examined prior to resection as an IgG4 autoimmune fibrosclerosing pseudotumour had not been suspected.

## 3. Discussion

IgG4-related autoimmune fibrosclerosing pseudotumours have been documented in a variety of different anatomical sites including the liver [2], lungs [3], and pituitary gland [4]. We are the first to report its occurrence in the stomach, a novel site for this pseudotumour subtype. These lesions were originally documented in patients with autoimmune pancreatitis (AIP), a disease first proposed by Yoshida in 1995 [5]. In this condition levels of serum IgG4 are significantly increased. There is marked infiltration of plasma cells and CD4- or CD8-positive T lymphocytes with fibrosis in the pancreas. To meet the histological diagnosis, there should be 10 or more IgG4-positive lymphoplasmacytic cells per high-power field [6]. In addition, extrapancreatic lesions occur in AIP such as sclerosing cholangitis, sclerosing sialadenitis, and retroperitoneal fibrosis which also show infiltration of IgG4-positive plasma cells. These extrapancreatic manifestations have led recently to some authors proposing the existence of a new clinicopathological entity, “Hyper-IgG4”or “IgG4-related sclerosing disease” [7, 8]. They have shown elevation of IgG4 within the organs affected although the degree of IgG4 staining is typically not directly reflective of serum IgG4 levels. Indeed in our patient, serum IgG4 levels were normal weeks after resection of the pseudotumour although levels could conceivably been elevated prior to surgery.

Histologically, inflammatory pseudotumours are characterised by an irregular proliferation of myofibroblasts with a surrounding inflammatory cell infiltrate, composed predominantly of T lymphocytes and plasma cells. The IgG-related inflammatory pseudotumours are characterised by dense infiltration of IgG4-positive plasma cells and lymphocytes associated with fibrosis. However, the mechanism by which increased IgG4 levels can induce pseudotumour formation is unclear, and typically the pseudotumour presents as the sole systemic manifestation of “Hyper IgG4”disease. 

Such IgG4-related inflammatory pseudotumours have been most commonly reported in the lungs with only occasional reports of extrapulmonary locations. Nine cases of lung IgG4-related pseudotumours were described by Zen et al. [3], 8 of which underwent surgical resection but one was successfully treated with corticosteroids alone. In another report one case of inflammatory pseudotumour of the left breast presented as a poorly defined 1.6 cm mass which was removed by excision biopsy due to diagnostic uncertainty [9]. 

To conclude, we present the first case report of a patient presenting with an IgG4-related pseudotumour within the stomach lining. There was no evidence of any other organ involvement in this patient. 

This case highlights how such a lesion can masquerade as another type of tumour, in this case a GIST, and as such, although rare, ought to be considered as part of the differential diagnosis for spindle cell lesions. The histopathological entity of IgG4-autoimmune fibrosclerosing pseudotumour is becoming recognised more frequently in a variety of locations, requiring treatment with steroids rather than surgery.

## Figures and Tables

**Figure 4:**
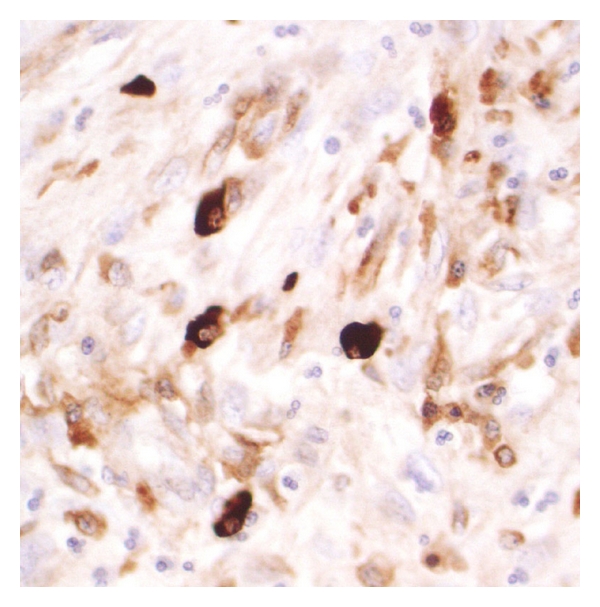
Histopathological analysis at 40x. IgG4 immunohistochemistry demonstrating IgG4 positive plasma cells.
